# Cannabinoid Receptors Modulate Excitation of an Olfactory Bulb Local Circuit by Cortical Feedback

**DOI:** 10.3389/fncel.2018.00047

**Published:** 2018-03-02

**Authors:** Frederic Pouille, Nathan E. Schoppa

**Affiliations:** ^1^Department of Physiology and Biophysics, University of Colorado Anschutz Medical Campus, Aurora, CO, United States; ^2^Neuroscience Program, University of Colorado Anschutz Medical Campus, Aurora, CO, United States

**Keywords:** olfaction, olfactory bulb, granule cell, mitral cell, corticofugal, cannabinoids

## Abstract

Recent studies have provided evidence that corticofugal feedback (CFF) from the olfactory cortex to the olfactory bulb (OB) can significantly impact the state of excitation of output mitral cells (MCs) and tufted cells (TCs) and also modulate neural synchrony. Interpreting these effects however has been complicated by the large number of cell targets of CFF axons in the bulb. Within the granule cell layer (GCL) alone, CFF axons target both GABAergic granule cells (GCs) as well as GABAergic deep short-axon cells (dSACs) that inhibit GCs. Because GCs are a major source of inhibition of MCs/TCs, CFF could be inhibitory to MCs (by exciting GCs) or disinhibitory (by exciting dSACs that inhibit GCs). In this study, we used patch-clamp recordings combined with optogenetic and electrical stimulation methods to investigate the role of presynaptic cannabinoid receptors in regulating CFF pathways, which could alter the weights of inhibition and disinhibition. Recording first from dSACs, we found that the cannabinoid receptor (CB-R) agonist WIN-55212.2 (WIN) reduced excitatory post-synaptic currents (CFF-EPSCs) driven by stimulation of CFF axons. The effects were reversed by the Type 1 CB-R (CB_1_-R)-specific antagonist SR-141716A. Furthermore, prolonged 5-s depolarizations applied to postsynaptic dSACs effectively reduced CFF-EPSCs in a CB_1_-R-dependent fashion, providing evidence for depolarization-induced suppression of excitation (DSE) at CFF-to-dSAC synapses. Further analysis indicated that CB_1_-Rs mediate widespread suppressive effects on synaptic transmission, occurring at CFF synapses onto different dSAC subtypes and CFF synapses onto GCs. Feedforward excitation of dSACs, mediated by MCs/TCs, however, was not impacted by CB_1_-Rs. In recordings from MCs, performed to examine the net effect of CB_1_-R activation on GC-to-MC transmission, we found that WIN could both increase and decrease disynaptic inhibition evoked by CFF axon stimulation. The exact effect depended on the size of the inhibitory response, reflecting the local balance of dSAC vs. GC activation. Our results taken together indicate that CB_1_-Rs can bidirectionally alter the weighting of inhibition and disinhibition of MCs through their effects on CFF pathways.

## Introduction

In mammals, the olfactory bulb (OB) processes sensory information contained within the activity of olfactory sensory neurons (OSNs) and passes its output through the axons of mitral cells (MCs) and tufted cells (TCs) to different olfactory cortical structures. At the same time, glutamatergic pyramidal cells in the olfactory cortex send dense feedback axonal projections to the OB, where they target MCs/TCs as well as a variety of GABAergic interneurons (Shipley and Adamek, 1984; Balu et al., 2007; Laaris et al., 2007; Matsutani, 2010; Boyd et al., 2012, 2015; Markopoulos et al., 2012; Rothermel and Wachowiak, 2014; Mazo et al., 2017). This feedback is derived from both anterior piriform cortex (aPC) and anterior olfactory nucleus (AON). *In vivo* studies have provided evidence that the excitatory corticofugal feedback (CFF) axons play an active role in determining the odor-evoked output of the OB and odor-driven behavior (Gray and Skinner, 1988; Martin et al., 2004; Kay and Beshel, 2010; Boyd et al., 2012; Otazu et al., 2015; Aqrabawi et al., 2016).

**Figure 1:**
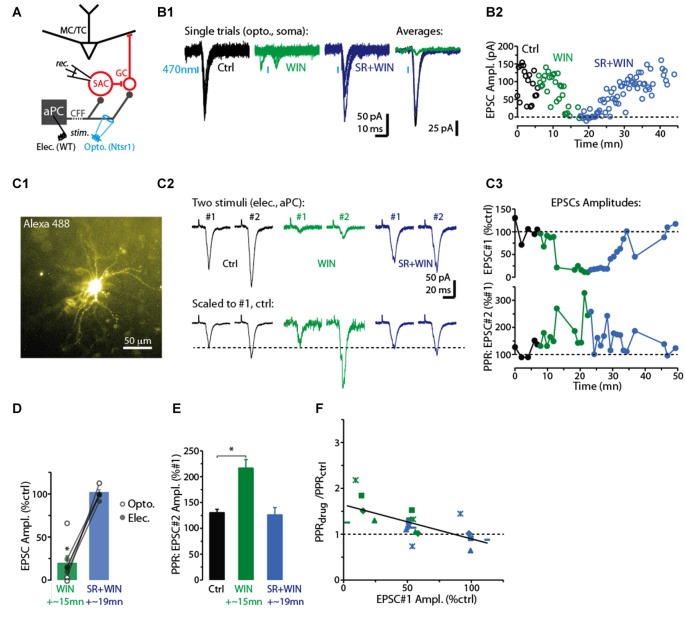
CB-R agonist and antagonist modulate corticofugal feedback (CFF)-EPSCs in deep short axon cells (dSACs). **(A)** Olfactory bulb (OB) circuit and experimental paradigm. The output mitral and tufted cells (MC/TCs) receive dendrodendritic input from GABAergic granule cells (GCs), which are themselves inhibited by GABAergic synapses from dSACs. CCF axons terminate on both GCs and dSACs. In experiments elsewhere in this figure, currents were recorded in dSACs (*V*_hold_ = −75 mV) while stimulating CFF axons. Stimulation was conducted either electrically in the anterior piriform cortex (aPC) in wild-type (WT) mice or with light (470 nm) pulses applied in the granule cell layer (GCL) of ntsr1-ChR2-YFP mice. **(B1)** Light-evoked (1 ms) current traces recorded from a dSAC. Left: overlaid trials (10 per condition) under control conditions (black), in the presence of WIN (10 μM; green), and in SR (10 μM) + WIN (blue). Right: overlaid mean CFF-EPSCs computed from left traces. Blue bars indicate time of 470 nm light pulse. Drugs were bath-applied. **(B2)** Time plot of the peak amplitude of the light-evoked CFF-EPSC for the entire experiment in **(A1)** (one data point per trial). **(C)** Test of CB_1_-R activation on the paired pulse ratio (PPR) during responses to electrical stimulation in aPC (100 μs, 2 mA). Illustrated is an epi-fluorescence image of the test dSAC (**C1**; located in the GCL), CFF-EPSCs evoked by a pair of electrical stimuli separated by 250 ms (**C2**; each trace = average of 12 trials), and the time-course of EPSC amplitude and PPR for this experiment (**C3**; each data point = average of 6 trials). The current traces in **(C2)** are shown in both unnormalized form (top) and normalized to the amplitude of the first EPSC (bottom). Note the marked WIN-induced increase in the PPR that coincides with a reduction in the amplitude of the first EPSC and the reversal by SR. **(D)** Summary of effects of WIN and SR+WIN on the amplitude of the CFF-EPSC. Bars reflect means ± SE of current remaining relative to control, measured over 4 min under each condition; superimposed data points are values for individual experiments. WIN data reflect seven recordings, four with electrical stimulation and three optogenetic. SR+WIN data reflect five recordings. *p < 0.02. **(E)** Summary of PPR measurements, showing an increase in PPR in WIN that was reversed by SR. Data reflect the five experiments in which both WIN and SR+WIN conditions were sampled. *p = 0.015, paired *t*-test. **(F)** The effects of WIN and SR+WIN on the PPR of the CFF-EPSC (Y-axis: PPR_drug_/PPR_ctrl_) were correlated with the drug effects on the amplitude of the first EPSC (X-axis). Each data point reflects one of the five experiments summarized in Part **(E)**. There are more data points (8 per condition) than number of experiments, reflecting the fact that in all experiments, time points at which the WIN or SR+WIN effects were only partially in effect were also sampled. Line: linear regression fit, *R*^2^ = 0.43, *p* < 0.002, *n* = 16).

Certainly the best-studied CFF pathway in the bulb involves contacts onto GABAergic granule cells (GCs; Shipley and Adamek, 1984; Balu et al., 2007; Laaris et al., 2007; Matsutani, 2010; Boyd et al., 2012; Markopoulos et al., 2012; see Figure 1A), which can shape MC activity through dendrodendritic inhibitory inputs. CFF axons from aPC also contact deep short axons cells (dSACs), which are GABAergic cells located in more inner regions of the OB, some of which can directly inhibit GCs (Pressler and Strowbridge, 2006; Eyre et al., 2008). In fact, CFF axons appear to make many more contacts on dSACs than on GCs (Boyd et al., 2012). The dual targeting of CFF axons onto both GCs and dSACs that inhibit GCs suggests that CFF axons have the capacity to fine-tune the level of GC-mediated inhibition of MCs as long as mechanisms are in place that can regulate one or the other CFF pathway. Some evidence for such modulation via neurotransmitters now exists. For example, GABA-mediated activation of presynaptic GABA_B_ receptors can depress synaptic transmission from CFF axons onto GCs (Mazo et al., 2016). Also, Type 1 cannabinoid receptors (CB_1_-Rs) are abundantly expressed on CFF axon terminals and can mediate a reduction in excitatory field potentials in the granule cell layer (GCL) by exogenous application of a CB-R agonist (Soria-Gómez et al., 2014). These observations are consistent with presynaptic effects of CB_1_-Rs on glutamatergic transmission (Kreitzer and Regehr, 2002; Kano et al., 2009; Araque et al., 2017) at CFF axon contacts onto GCs and/or dSACs. Top-down neuromodulation of the CFF pathways is also possible, for example through cholinergic or noradrenergic projections into the GCL (Záborszky et al., 1986; McLean et al., 1989).

In this study, we used patch-clamp recordings in OB slices combined with electrical and optogenetic stimulation methods to further assess the function of the cannabinoid receptor system in regulating CFF feedback pathways in OB. Building on the prior work of Soria-Gómez et al. (2014), we sought to examine which specific CFF pathway(s) are modulated by CB_1_-Rs, and also whether endogenous cannabinoids, the endocannabinoids, can activate the receptors. Much of our focus was on the CFF axon-to-dSAC pathway, since at least the most common subclass of dSACs (known as Blanes cells; Eyre et al., 2008) can undergo long-lasting depolarizations and spike activity (Pressler and Strowbridge, 2006) that in other systems have been shown to be required for the release of endocannabinoids (Kreitzer and Regehr, 2001; Ohno-Shosaku et al., 2002; Kano et al., 2009). We also evaluated CB_1_-R-mediated effects at CFF axon-to-GC synapses and the net effect of CB_1_-R activation on disynaptic inhibition of MCs evoked by stimulation of CFF axons (Balu et al., 2007; Markopoulos et al., 2012).

## Materials and Methods

All protocols and experiments involving vertebrate animals were approved by the Institutional Animal Care and Use Committee at the University of Colorado Anschutz Medical Campus (UCAMC).

### Electrophysiological Recordings

Electrophysiological recordings were performed in acute horizontal slices (300–350 μm thick) of OB prepared from wild-type (WT) C57BL/6 mice (Charles River, Wilmington, MA, USA) of both sexes or one of two types of transgenic mice. These included neurotensin receptor 1 (ntsr1)-Cre recombinase (Cre)-channelrhodopsin-2 (ChR2)-YFP (Ntsr1-Cre^+/+^ and ChR2-YFP^+/–^ or ^+/+^, C57BL/6/129/Swiss/FVB mixed background) mice or olfactory marker protein (OMP)-ChR2-YFP (^+/–^ or ^+/+^, C57BL/6 background; stock OMP^tm1.1(COP4*/EYFP)Tboz^/J, Jackson Laboratory, Bar Harbor, ME, USA) mice at postnatal age 10–28 days, of both sexes. Ntsr1-ChR2-YFP mice were obtained by crossing Ntsr1-Cre mice (STOCK Tg(Ntsr1-cre)GN209Gsat/Mmucd, GENSAT Project, The Rockefeller University, New York, NY, USA) with loxP/ROSA-ChR2-YFP mice (C57BL/6;129S-Gt(ROSA)26Sor^tm32(CAG-COP4*H134R/EYFP)Hze^/J, Jackson Laboratory). While housed in the UCAMC animal facility, animals had full and continuous access to food and water. The preparation of bulb slices and genotyping was as previously described (Schoppa et al., 1998; Gire et al., 2012). In slice preparation, animals were anesthetized by inhalation of isoflurane prior to decapitation. Brain slices were prepared from 84 mice across all experiments.

Recordings were made at 27–31°C in artificial cerebrospinal fluid (ACSF) containing (in mM): NaCl 125, NaHCO_3_ 25, NaH_2_PO_4_ 1.25, KCl 3, CaCl_2_ 2 or 3, MgCl_2_ 0.5–1, and Glucose 25 (280–290 mOsm, pH 7.3), and was oxygenated (95% O_2_/5% CO_2_). Neurons were recorded using glass micropipettes (World Precision Instruments, Inc., Sarasota, FL, USA; 4–12 MΩ) filled most often with an internal solution containing (in mM): K-gluconate 147.5, EGTA 1, MgCl_2_ 2 or 6, CaCl_2_ 0.025, Na_2_-ATP 0.5, Na-GTP 0.5, HEPES 10 (280–290 mOsm, pH 7.3). Alexa Fluor 488 or 594 (5–10 μM) was sometimes added to the internal solution for further morphological assessment. In recordings of inhibitory currents in MCs conducted using a 0 mV holding potential, K-gluconate in the internal solution was replaced with equimolar cesium gluconate. All neurons were recorded in the whole-cell configuration in voltage-clamp mode.

Pharmacological agents were diluted in the ACSF stock perfusing the chamber, and included: cannabinoid receptor (CB-R) agonist WIN 55,212-2 (WIN, Tocris Bioscience, Bristol, UK), type-1 CB-R antagonist SR-141716A (SR, Tocris), GABA_A_ receptor antagonist SR-95531/gabazine (GBZ, Tocris), AMPA receptor antagonist 2,3-dihydroxy-6-nitro-7-sulfamoyl-benzo[f]quinoxaline-2,3-dione (NBQX; Sigma-Aldrich, St. Louis, MO, USA) and NMDA receptor antagonist D-(-)-2-Amino-5-phosphonopentanoic acid (D-AP5, Tocris).

Slices were mounted in a perfusion chamber under an Axioskop 2 microscope (Carl Zeiss, Thornwood, NY, USA) set-up with differential interference contrast (DIC) and epi-fluorescence optics. Epifluorescence was captured by an Axiocam HSm (Zeiss) camera; images were acquired using AxioVison software. Neurons were visually identified based on the location and size of their soma, and, in a subset of experiments, neuron identity was confirmed by intracellular filling with Alexa Fluor. Typically, deep short-axon cells (dSACs) had significantly larger cell bodies (diameter ≥ 20 μm) than GCs (diameter ≤ 15 μm); GCL-dSAC cell bodies were located between GC islets. GCs had a long apical dendrite perpendicular to the MC layer, whereas dSACs had multiple main dendrites, mostly parallel to the internal plexiform layer (IPL) in the case of IPL-dSACs, and more stellate for GCL-dSACs. In a few cases, IPL-dSACs also revealed an axon projecting to the glomerular layer (GML).

All recordings were performed using a Multi-Clamp 700B amplifier (Molecular Devices, Sunnyvale, CA, USA) and a Digidata 1322A digital interface (Axon Instruments, Union City, CA, USA); data were acquired (10 kHz digitization; 4 kHz low-pass filtered using an eight-pole Bessel filter) and analyzed in AxoGraph X software or on a Macintosh G5. Recording sessions with access resistance higher than 15 MΩ (for MCs and SACs) and 20 MΩ (for GCs) were discarded. Reported holding potential (*V*_hold_) values were not corrected for liquid junction potentials.

### Stimulation Protocols

To recruit ChR2-expressing corticofugal (CFF) axons in slices prepared from ntsr1-ChR2-YFP mice, 1–5 ms blue-light square-pulses (470 nm high-power LED, Thorlabs, Inc., Newton, NJ, USA) were delivered through a 40× objective focused and centered on either the cell bodies of the test cells (for recordings in dSACs or GCs) or 250–300 μm away in the GCL in recordings of MCs (MCs; to coincide with the peak in the diffuse YFP signal in CFF axons). Light stimuli were given at 30–60 s intervals.

To electrically stimulate CFF axons in WT mice, the tip of a tungsten bipolar electrode was placed in the layer 2/3 of the most anterior part of the aPC, 2–3 mm from the posterior border of the OB. Biphasic current-pulses (1–4 mA; one 100 μs stimulus, five stimuli at 40 Hz, or 10 stimuli at 100 Hz) were delivered via a stimulus isolator (World Precision Instruments, Inc., Sarasota, FL, USA). Stimuli were applied every 10–20 s. For electrical stimulation of the aPC, we typically chose the most ventral slices as they were more likely to retain CFF axons un-severed from the aPC. We then selected slices in which aPC stimulation could evoke a local field potential signal, as recorded by a glass micropipette filled with ACSF and lowered in the GCL (resistance 5–7 MΩ). A cut through the lateral olfactory tract (LOT) was made about 0.6 mm from the posterior border of the OB in order to prevent antidromic stimulation of the OB.

Axons of OSNs were stimulated either by using 470 nm light illumination of the olfactory nerve (ON)/GMLs in slices prepared from OMP-ChR2-YFP animals. Alternatively, a stimulating electrode was placed in the ON layer ~50 μm from the GML in WT mice; single stimulus pulses (100 μs; 50–250 μA) were applied.

In both dSACs and GCs, we attempted to produce depolarization-induced suppression of excitation (DSE) or inhibition (DSI) by using a 5-s voltage step command from −75 mV (when monitoring evoked excitation) or from ~–45 mV (when monitoring evoked inhibition) to 0 mV, in neurons recorded in voltage-clamp mode. Pre-depolarization and post-depolarization afferent stimulation were respectively given 52.5 s before and 2.5 s after the end of the 5 s voltage step to 0 mV in most experiments. This ensured a constant 60 s interval between all stimuli and helped prevent run-down of the ChR2 current.

### Analysis of Electrophysiological Recordings

The paired-pulse ratio (PPR) of synaptic responses evoked by two stimuli separated by 250 ms was computed as the peak amplitude of the mean post synaptic current (PSC) evoked by the second stimulus, divided by the peak amplitude of the mean PSC evoked by the first stimulus (mean PSCs obtained by averaging 12–24 trials over 4 min). The change in the PPR of the CFF-EPSCs induced by WIN or SR (PPR_drug_/PPR_ctrl_) was computed as the PPR of the CFF-EPSC during drug application divided by the PPR of the CFF-EPSC during the control period. The change in the PPR of the CFF-EPSCs induced by the 5 s depolarization (PPR_post_/PPR_pre_) was computed as the PPR of the CFF-EPSC evoked 2.5 s after the end of the depolarization divided by the PPR of the CFF-EPSC evoked before the depolarization.

To monitor the number of discreet IPSCs observed in the inhibitory currents evoked in MCs by a stimulation of the CFF pathway, we ran an event detection algorithm (in Axograph) within a time window chosen containing most of the evoked response. For MCs tested in WIN, the long stimulation used (5 stimuli at 40 Hz or 10 stimuli at 100 Hz) led us to choose a relatively large detection window (250 ms-duration, starting at the first stimulus). When testing the effect of low concentrations of GBZ on the probability of evoking an IPSC in MCs with CFF stimulation, when the failure rate was relatively high, we restricted detection to a shorter time window (50 ms-duration). For those MCs in which GBZ or WIN induced an increase in the number of detected IPSCs, we computed for each MC the mean number of detected IPSCs (averaged over 4 min) at the peak of the increase or when the increase plateaued. For dSACs in which stimulation of the CFF pathway evoked a biphasic EPSC-IPSC sequence, the average EPSC was recorded in isolation at −75 mV, and then scaled to the average EPSC-IPSC sequence recorded at −45 mV until the initial slopes of the two traces overlapped. The scaled EPSC trace was then subtracted from the EPSC-IPSC trace, in order to isolate the IPSC component and measure its peak amplitude.

### Statistics

Data in the text and figure plots are reported as mean ± standard error of the mean (SEM). Unless otherwise noted, non-parametric statistical tests were used in the analysis, either the Mann-Whitney U test or the Wilcoxon Signed-Rank test (for paired samples). Student’s *t*-test (two-tailed with equal or unequal variance, as appropriate) was used for data that appeared to be distributed normally or if *n* values were greater than 50. Statistical tests were performed in Microsoft Office Excel.

## Results

For studying the role of cannabinoid receptors (CB-Rs) in modulating CFF onto deep short-axon cells (dSACs) and GCs in the bulb, two approaches were used to activate CFF axons (Figure 1A). The first was 470 nm LED light pulses (1–5 ms) applied in slices prepared from neurotensin receptor-1-channelrhodopsin-2-YFP (ntsr1-ChR2-YFP) mice (see “Materials and Methods” section), which selectively express ChR2 in pyramidal neurons of the olfactory cortex. In these optogenetic experiments, light pulses were applied to specific locations through a 40× objective. Alternatively, CFF axons were activated with electrical stimulation of pyramidal cells in Layer 2/3 of the aPC in WT mice (1–4 mA, 100 μs pulses; Balu et al., 2007). Each method had value for our study. Electrical stimulation enabled us to recruit a sub-population of CFF axons that originated in aPC, different from optogenetic stimulation, which likely activated CFF axons from both aPC and AON. Thus, the method had higher specificity, relevant for example because it is not known whether CFF axons from AON even target dSACs. On the other hand, local optogenetic stimulation of CFF axons enabled us to perform specific pharmacological analyses with less concern that drug effects were due to changes in neural excitability in aPC.

In voltage-clamp recordings from dSACs (*V*_hold_ = −75 mV), the two stimulation methods elicited kinetically similar excitatory postsynaptic currents (EPSCs) that were consistent with monosynaptic, AMPA receptor-mediated excitatory transmission (Figure 1B1). Light-evoked EPSCs had onset-delays that depended on the duration of the light pulse, being very short for 1-ms light-pulses (time after start of light pulse = 2.8 ± 0.1 ms; *n* = 4) and somewhat longer for 5-ms light pulses (6.5 ± 1.0 ms, *n* = 18). The longer onset-delays for 5-ms light pulses were expected for monosynaptic EPSCs since much of the glutamate release should be associated with axon terminal repolarization. EPSCs evoked by electrical stimulation had relatively long onset-delays near 10 ms (9.6 ± 0.5 ms, *n* = 18; Figure 1C2), likely reflecting the distance between aPC and the test cells in the bulb (2–3 mm). The EPSCs evoked by the two stimulation methods had similar kinetics for rise-time (10%–90% rise-time = 1.9 ± 0.3 ms for light; 2.5 ± 0.3 ms for electrical) and decay (time-constant = 3.1 ± 0.3 ms for light; 3.6 ± 0.5 ms for electrical; *p* > 0.1, *n* = 18 for each EPSC type).

### CB_1_-R-Mediated Modulation of the Excitatory Synapses Between CFF Axons and dSACs

Since Type 1 cannabinoid receptors (CB_1_-Rs) are expressed in CFF axons in the OB (Soria-Gómez et al., 2014), we assessed whether CFF axons that contact dSACs are modulated by CB_1_-R activation. Bath perfusion of the CB-R agonist WIN 55,212-2 (WIN; 10 μM) reduced the amplitude of light- and electrically-evoked CFF-EPSCs (by 71 ± 11%; control amplitude: −78 ± 16 pA; WIN: −14 ± 4 pA; *p* < 0.02, *n* = 7; two examples in Figures 1B1–C3, summary in Figure 1D), while subsequent perfusion of a solution that contained both WIN and the CB_1_-R-specific antagonist SR 141716A (SR; 10 μM) fully reversed the WIN effect (EPSC amplitude in SR+WIN = 100 ± 3% of control; *p* > 0.2, *n* = 5; Figures 1B–D). These results indicate that activation of CB_1_-Rs potently reduces CFF-EPSCs evoked in dSACs.

A change in the PPR of postsynaptic currents evoked by a pair of stimuli is typically taken to reflect a change in the presynaptic probability of transmitter release. Here we tracked the PPR of CFF-EPSCs that were evoked by electrical stimulation (the PPR of light-evoked EPSCs could be distorted by desensitization of the ChR2 current in CFF axons). With two stimuli separated by 250 ms, the decrease of the CFF-EPSC in WIN was associated with a 60 ± 12% increase in the PPR (amplitude ratio of second EPSC vs. first EPSC; *p* = 0.015 in paired *t*-test, *n* = 5; Figures 1C2,C3,E). The greater PPR in WIN would be expected if the smaller WIN-evoked CFF-EPSC in response to the first stimulus were due to a lower probability of release; this would make more transmitter-containing vesicles available for release for the second stimulus. Also, as expected for CB_1_-R-mediated effects on release probabilities, SR recovered control-like levels of PPR (1.19 ± 0.14; *p* = 0.7 in paired *t-test*, *n* = 5). Furthermore, across all experiments we found proportional effects of the drugs on the amplitude of the first EPSC and the change in PPR (linear regression of the relationship between the two: *R*^2^ = 0.43, *p* < 0.002, *n* = 16; Figure 1F).

### CB_1_-R-Mediated DSE at CFF-to-dSAC Excitatory Synapses

**Figure 2:**
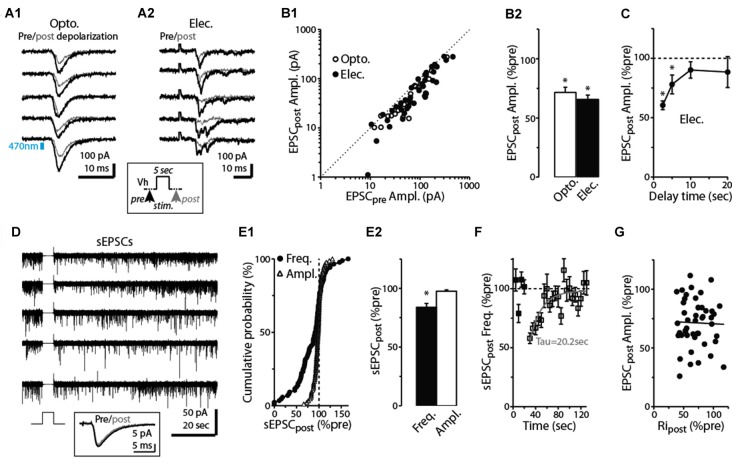
Prolonged depolarization of postsynaptic dSACs reduces CFF-EPSCs. **(A1)** Light-evoked CFF-EPSCs recorded during five consecutive trials from dSACs that were exposed to a 5-s depolarization to 0 mV. Black traces (“Pre”) reflect CFF-EPSCs evoked prior to the depolarization; gray (“post”) reflects EPSCs evoked 2.5 s after the depolarization. The boxed inset at bottom illustrates the protocol. **(A2)** Results from a second, similar recording in which CFF-EPSCs were evoked by electrical stimulation in aPC. **(B1)** Summary of 61 recordings from dSACs in which the EPSC evoked after the 5-s depolarization is plotted vs. the EPSC recorded prior to the depolarization. Nearly all of the data points lie below unity (dashed line), indicating depolarization-induced suppression. Open circles: light-stimulation in ntsr1-ChR2-YFP slices, *n* = 18; filled circles: electrical stimulation in WT slices, *n* = 43. Each data point reflects one dSAC recording (averages of 3–23 trials per condition per cell). **(B2)** Prolonged depolarization of the dSAC significantly reduced CFF-EPSCs evoked by both light (left) and electrical stimulation (right). Data are from part **(B1)**. *p < 0.001. **(C)** Values for mean ± standard error of the mean (SEM; *n* = 7) for the normalized CFF-EPSC recorded at various time intervals after the end of the 5-s depolarization. Note that the depolarization-induced reduction in the CFF-EPSC was mainly over by 10 s after the end of the depolarization. *p ≤ 0.014, paired *t*-test, *n* = 7. **(D)** Five consecutive current traces (band-pass filtered at 0.01–10 kHz) recorded from a dSAC in which the effect of a 5-s depolarization was tested on spontaneous EPSCs (sEPSCs). Note the decrease and then recovery of the sEPSCs following the depolarizations. The boxed inset at bottom includes the averaged sEPSCs recorded before and just after the depolarization for this experiment. **(E1)** Summary from 86 dSAC recordings in which the effect of the depolarization of the test dSAC on sEPSCs was tested, plotted as a cumulative distribution of normalized sEPSC frequency and amplitude. Vertical dashed line: no depolarization-induced suppression of excitation (DSE). Each point reflects one dSAC. **(E2)** Summary of depolarization effects on sEPSC frequency and amplitude plotted as histograms. *p = 0.01, paired *t*-test, *n* = 86. **(F)** The effect of dSAC depolarization on sEPSC frequency plotted as a function of time after the end of depolarization. Results reflect 48 experiments in which sEPSC frequency measured 5 s after the end of the depolarization was ≤ 90% of control. Gray line: mono-exponential regression of the average data points (time constant = 20.2 s). **(G)** Effect of the 5-s depolarization on the CFF-EPSC plotted as a function of the effect on cell input resistance (*Ri*_post_). Measurements, occurring before and 2.25 s after the end of the depolarization, reflect 48 experiments as in Part **(F)**. Line: linear regression fit, *R*^2^ = 0.0008, *p* = 0.8).

At many excitatory synapses, sustained depolarization of a postsynaptic neuron can cause retrograde activation of presynaptic CB_1_-Rs leading to decreases in glutamate release (Kreitzer and Regehr, 2001; Ohno-Shosaku et al., 2002; Kano et al., 2009). This phenomenon, known as DSE, is driven by endocannabinoids, anandamide and 2-arachidonylglycerol, released from the postsynaptic cell. Because prolonged depolarization and persistent spiking are features inherent to at least some dSACs (Pressler and Strowbridge, 2006), we tested the effect of a 5-s depolarization (to 0 mV) applied to the dSAC on the CFF-EPSC. In slices prepared from ntsr1-ChR2-YFP mice, light-induced CFF-EPSCs evoked 2.5 s after the end of the depolarization were reduced by 29 ± 4% vs. CFF-EPSCs evoked prior to the depolarization (*p* < 0.001, *n* = 18; Figures 2A1,B1,B2). A similar reduction due to prolonged depolarization (34 ± 4% decrease, *p* < 0.001, *n* = 43) was observed for CFF-EPSCs evoked by electrical stimulation (Figures 2A2–B2). Increasing the time delay between the end of the 5-s depolarization and the test stimulus revealed that the suppression of the CFF-EPSC mainly ended by 10 s after the depolarization (*p* > 0.1, *n* = 7, Figure 2C). Somewhat more precise information about the time-course of the DSE was obtained from recordings of spontaneous EPSCs (sEPSCs) in dSACs. The 5-s depolarization reduced the frequency of sEPSCs by 16 ± 5% (*p* = 0.01 in paired *t*-test, *n* = 86; Figures 2D–E2); this suppression decayed with a time constant of 20 s (Figure 2F). These measured time-courses for DSE at CFF-to-dSAC synapses are within the range observed for CB_1_-R-mediated DSE at other synapses (Kreitzer and Regehr, 2001; Ohno-Shosaku et al., 2002). The 5-s depolarization did not alter the amplitude of the sEPSCs (Figure 2D *inset*, Figures 2E1,E2; *p* = 0.25 in paired *t*-test, *n* = 86).

**Figure 3:**
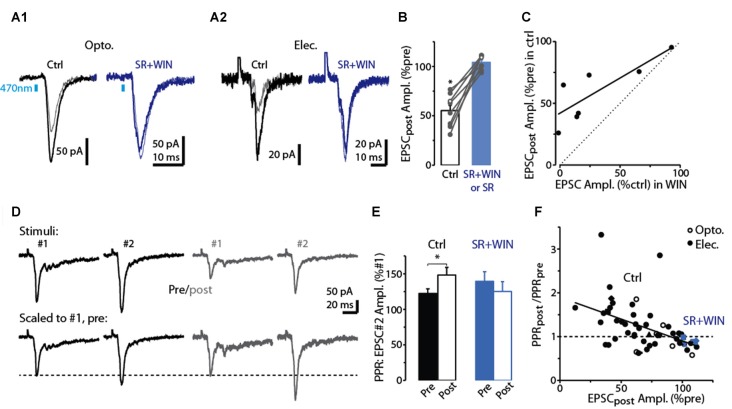
Evidence for DSE mediated by presynaptic CB_1_-Rs in dSACs. **(A1)** Light-evoked CFF-EPSCs recorded from a dSAC prior to (darker traces) and 2.5 s after a 5 s depolarization (lighter traces) under control conditions (left) and in the presence of the CB_1_-R antagonist SR (right; SR solution contains WIN). Traces reflect means of 3–10 trials. **(A2)** Results from a second experiment like **(A1)** except that the CFF-EPSCs were evoked by electrical stimulation in aPC. **(B)** Summary from eight experiments similar to **(A1,A2)** showing that SR (*n* = 4) or SR+WIN (*n* = 4) fully reverses the effect of the depolarization on the CFF-EPSC. Filled symbols: electrical stimulation; open symbols: light stimulation. *p < 0.02, *n* = 8. **(C)** Comparison of the effects of prolonged depolarization of the test dSAC vs. application of the CB_1_-R agonist WIN on CFF-EPSCs (in the absence of depolarization). Each data point reflects one of seven recordings in which both were monitored. Line: linear regression fit, *R*^2^ = 0.67, *p* < 0.02, *n* = 7). Dotted line: unity. **(D)** CFF-EPSCs in a dSAC evoked by paired electrical stimulation recorded before (left, darker traces) and 2.5 s after (right) a 5-s depolarization. Top traces: averages of 10 trials; bottom: same traces scaled to the peak amplitude of the first EPSC. Note the increase in PPR for EPSCs evoked following the depolarization. The two-stimulus bursts used to estimate PPR before and after the depolarization were separated by 250 ms in these experiments. **(E)** Summary of effects of prolonged depolarization on PPR. Results reflect 43 experiments in which PPRs were measured before and after the depolarization under control conditions (left) and four experiments in which the same measurements were made in SR+WIN (right). *p = 0.012 in paired *t*-test, *n* = 43. **(F)** The effect of the 5-s depolarization on PPR of the CFF-EPSC_post_ (Y-axis: “PPR_post_ /PPR_pre_”) was correlated with its effect on the peak amplitude of the first EPSC. Each data point reflects a single experiment (averages of 3–23 trials) conducted under control conditions (black points) or in SR+WIN (blue points). Line: linear regression of the combined control and SR+WIN data (*R*^2^ = 0.23, *p* < 0.001, *n* = 43).

As expected if the reduction in the CFF-EPSC induced by prolonged depolarization of dSACs was mediated by presynaptic CB_1_-Rs, we found that the CB_1_-R-specific antagonist SR largely eliminated the reduction (Figures 3A,B; 3.9 ± 2.1% increase in CFF-EPSC after 5-s depolarization in SR, *p* = 0.31 in paired *t*-test, *n* = 8). In addition, the depolarization-induced reduction in the CFF-EPSC was associated with a 26 ± 8% increase in the PPR (*p* = 0.012 in paired *t*-test comparing PPR before and after the 5-s depolarization, *n* = 43; Figures 3D,E). Further supporting presynaptic effects mediated by CB_1_-Rs, the depolarization-induced change in PPR was modestly but significantly correlated with the change in the amplitude of the first CFF-EPSC (*R*^2^ = 0.23, *p* < 0.001, *n* = 43; Figure 3F). It should be noted that in the experiments with SR involving prolonged depolarization of dSACs, the solution containing SR also included the CB-R agonist WIN in 4 of 8 experiments (see “Discussion” section). In the four experiments in which SR was applied without WIN, SR clearly eliminated the depolarization-induced reduction in the CFF-EPSC (0.4 ± 3.2% increase after 5-s depolarization in SR, *p* = 0.64 in paired *t*-test) that occurred under control conditions (44 ± 8% decrease in CFF-EPSC after 5-s depolarization, *p* = 0.014 in paired *t-test*).

**Figure 4:**
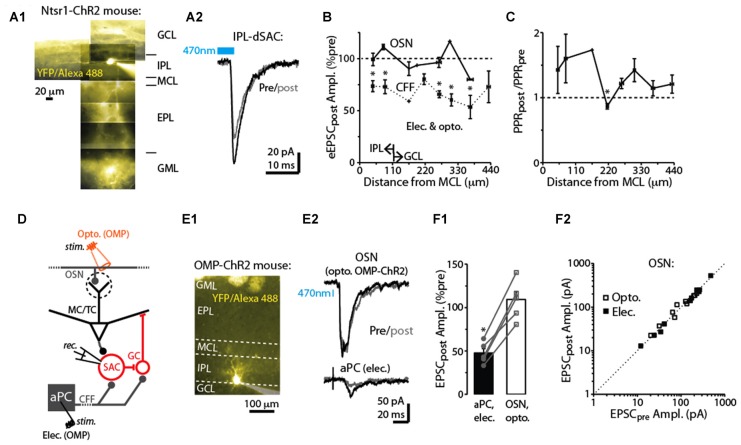
Specificity of DSE in dSACs. **(A1)** Reconstructed epi-fluorescence image of an internal plexiform layer (IPL)-dSAC filled with Alexa 488 in a bulb slice from an ntsr1-ChR2-YFP mouse. Note dendrites restricted to the IPL and processes crossing through the external plexiform layer (EPL). Signals in the glomerular layer (GML) and GCL likely in part reflect ChR2-YFP. MCL = mitral cell layer. **(A2)** Mean light-evoked CFF-EPSCs (averages of 10 trials) recorded from the IPL-dSAC in **(A1)**, both before (black) and 2.5 s (gray) after a 5 s depolarization to the test cell. **(B)** Summary of the effect of a 5-s depolarization of the test dSAC on the amplitude of EPSCs plotted vs. the distance between the dSAC cell body and the MCL. Note that for the CFF-EPSCs (*n* = 61 dSACs), the magnitude of DSE is independent of the location of the dSAC. Also plotted is a similar analysis performed on EPSCs evoked by stimulation of olfactory sensory neurons, OSNs (*n* = 21 dSACs; see example in Part **E**). Bins: 55 μm. The IPL/GCL border was located approximately 110 μm from the MCL (arrows). *p < 0.05, *n* = 7–14. **(C)** Summary of the effect of dSAC depolarization on the PPR of CFF-EPSCs vs. distance between the dSAC cell body and the MCL. Same experiments as CFF data in Part **(B)**. *p = 0.022, paired *t*-test, *n* = 7. **(D)** Protocol for comparing CCF-EPSCs in dSACs with feedforward EPSCs evoked by stimulation of OSN inputs. Feedforward EPSCs were evoked using light pulses in olfactory marker protein (OMP)-ChR2-YFP mice (as shown) or with electrical stimulation of OSNs. **(E)** Feedforward EPSCs evoked in a dSAC were not impacted by dSAC depolarization. Illustrated is the image of the Alexa Fluor-filled IPL-dSAC **(E1)** and current traces in the same cell evoked by optogenetic OSN stimulation (**E2**, top) and electrical stimulation in aPC (**E2**, bottom). In the image in **(E1)**, note the fluorescent glomeruli at top reflecting ChR2-YFP. **(F1)** In five dSACs in which both OSN-driven excitatory currents and CFF-EPSCs were recorded, depolarization of the test cell reduced the CFF-EPSC without impacting the OSN-driven current. Five to 20 trials were recorded in each cell for each type of stimulus. *p < 0.001, paired *t*-test, *n* = 5. **(F2)** Effect of dSAC depolarization on OSN-driven excitatory currents in 20 dSACs. Experiments were separated by whether currents were light evoked (in OMP-ChR2 mice; open symbols) or by electrical stimulation in the OSN layer (filled symbols).

The pharmacological analysis and PPR measurements support that the reduction in the CFF-EPSC following a 5-s depolarization in the test dSACs is due to CB_1_-R-mediated reductions in glutamate release from CFF terminals, but we also considered one other possible explanation. The 5-s depolarization did significantly decrease the input resistance of dSACs (30 ± 3% reduction, *p* < 0.001, *n* = 47; 379 ± 47 MΩ pre- vs. 236 ± 26 MΩ post-depolarization; measured 250 ms before each CFF stimulus), which in principle could have contributed to part of the reduction in the CFF-EPSC. However, arguing against a significant contribution for this mechanism was the fact that the magnitude of DSE was uncorrelated with the change in input resistance (Figure 2G). Furthermore, as we will show below (see Figures 4D–F), prolonged depolarization of the dSAC did not alter feedforward EPSCs in dSACs evoked by stimulation of sensory inputs even while it altered CFF-EPSCs. If changes in input resistance had been the cause of the depolarization-induced reduction in the CFF-EPSC, reductions should also have been observed for the feedforward EPSCs.

Across our sample of dSAC recordings in which we tested for DSE, we observed considerable variability in the degree of DSE (see values along *x*-axis in Figure 3F). Amongst the possible explanations include variability in the effectiveness of the 5-s depolarization in eliciting release of endocannabinoids. Alternatively, there could have been differences across recordings in the number of presynaptic CB_1_-Rs on the CFF axon terminals. Consistent with this latter explanation were the results from seven dSAC recordings in which we assessed both DSE and also the effect of the CB_1_-R agonist WIN on the CFF-EPSC. In these experiments, DSE and WIN effects were found to be highly correlated (*R*^2^ = 0.67, *p* = 0.024, *n* = 7; Figure 3C). Such a relationship would not have been expected if the number of presynaptic CB_1_-Rs was invariant and the variability was due to differences in endocannabinoid concentration. The same experiments also revealed that the magnitude of DSE was consistently smaller than the WIN-effect (CFF-EPSC reduced by 41 ± 9% due to DSE vs. 70 ± 12% by WIN, *p* < 0.05, *n* = 7). This would be expected if the endocannabinoids released by the dSACs did not saturate the CB_1_-Rs.

In the hippocampus, endocannabinoids released by a depolarized principal neuron can inhibit synaptic transmission onto a neighboring principal neuron (Wilson and Nicoll, 2001). During five simultaneous recordings from pairs of dSACs located within 27–60 μm of each other, we found evidence for such transneuronal effects in only one of the pairs (data not shown; 25% reduction in the CFF-EPSC in the test cell due to depolarization of other cell). Thus, trans-DSE between nearby dSACs appeared possible, but was uncommon.

### dSAC Subtype and Pathway Specificity of Depolarization-Induced Synaptic Changes

dSACs include several distinct subtypes of neurons that differ in location and axodendritic morphology (among other features; Pressler and Strowbridge, 2006; Eyre et al., 2008, 2009). For example, dSACs with cell bodies in the IPL project their axons into the GML of the bulb and have long dendrites that remain in the IPL, whereas dSACs in the GCL project axons into the GCL or external plexiform layer (EPL) and have stellate-like dendrites that are restricted to the GCL/EPL. We thus mapped the strength of DSE with the distance of each dSAC soma from the mitral cell layer. DSE of evoked CFF-EPSCs occurred in dSACs located in both the GCL (example in Figure 2A2) and IPL (examples in Figures 2A1, 4A1,A2) and remained similar in strength for dSACs at all locations (depolarization-induced decrease: 26 ± 4% for IPL-dSACs, *p* < 0.001, *n* = 20; 36 ± 4% in GCL-dSACs, *p* < 0.001, *n* = 41; *p* > 0.1 in comparison between DSE in IPL-dSACs and GCL-dSACs; Figure 4B). The fractional change in PPR following a 5-s depolarization was also invariant across dSAC subtype (Figure 4C). Thus, DSE had indistinguishable properties for different dSAC subtypes in our recordings.

In addition to being excited by CFF axons, dSACs can also be excited by feedforward signaling via TCs (Burton and Urban, 2015) and perhaps MCs. The DSE appeared however to be specific to the CFF pathway. EPSCs in dSACs that were evoked by electrical or optogenetic stimulation of OSN axons (Figure 4D; see “Materials and Methods” section), presumably reflecting feedforward inputs from TC/MCs, were not impacted by a 5-s depolarization applied to the test dSAC (1 ± 3% decrease in amplitude, *p* > 0.1, *n* = 20; Figures 4E1,E2,F2). The failure to elicit DSE in the feedforward path occurred for both dSACs in the GCL (7 ± 4% decrease following depolarization, *p* > 0.1, *n* = 9) and IPL (3 ± 4% increase, *p* > 0.1, *n* = 12; Figure 4B). The absence of DSE of feedforward EPSCs was not because these dSACs failed to release endocannabinoids. In 5 dSACs in which we were able to record responses to stimulation of both feedforward and CFF pathways, the CFF-EPSCs were markedly reduced following dSAC depolarization (47 ± 5% of control) even though the feedforward EPSC was unaffected (9 ± 9% increase; *p* = 0.01 in paired *t*-test comparison with CFF-EPSCs; Figures 4E1–F1).

**Figure 5:**
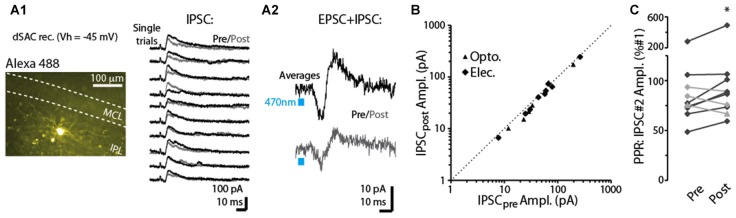
Depolarization-induced suppression of disynaptic IPSCs in dSACs. **(A1)** Right: outward currents recorded from an IPL-dSACs at *V*_hold_ = −45 mV. Illustrated are 10 consecutive trials in which currents evoked by electrical stimulation of the aPC were recorded before (black traces) and 2.5 s after (gray traces) a 5-s depolarization applied to the cell. Left: epi-fluorescence image of this IPL-dSAC. **(A2)** Light-evoked EPSC-IPSC sequences in a different, GCL-dSAC recorded before (top trace; average of 10 trials) and after (bottom) depolarization of the test cell. **(B)** Summary of depolarization-induced effects on disynaptic IPSCs recorded in 15 dSACs. Each data point reflects one experiment (5–10 trials per experiment). Note the consistent small effect, as seen in the offset of the data points from the diagonal line reflecting unity. **(C)** Summary of depolarization effects on the PPRs of the disynaptic IPSCs for 10 experiments. Black lines reflect 7 of the 10 experiments in which the depolarization reduced the amplitude of the IPSC. *p = 0.038, paired *t*-test, *n* = 7.

In a subset of dSACs voltage-clamped at a more depolarized potential (*V*_hold_ = −45 mV), stimulation of the CFF pathway evoked an inhibitory postsynaptic current (IPSC), either in isolation or along with an EPSC (see two example recordings in Figures 5A1,A2). This IPSC, which was blocked by the GABA_A_ receptor-blocker gabazine (10 μM; *n* = 3), occurred 3.1 ± 0.9 ms after the onset of the EPSC in recordings in which an EPSC-IPSC sequence was observed. The relative timing of the IPSC implies that it is disynaptically mediated, and suggests the possibility that dSACs targeted by CFF axons may be interconnected. The disynaptic IPSCs in dSACs, whether light- or electrically evoked, were, like CFF-EPSCs, reduced by a 5-s depolarization of the dSACs, although the effect was modest (13 ± 3% reduction in IPSC peak amplitude, *p* < 0.05, *n* = 15; Figures 5A,B). As observed above with the CFF-EPSC, the reduction in the IPSC was associated with an increase in PPR (14 ± 4% increase in PPR in 7 dSACs that showed depolarization-induced reduction in the CFF-IPSC amplitude, *p* = 0.038 in paired *t*-test; Figure 5C). The effect on the IPSC could be due either to depolarization-induced suppression of GABA release from cells inhibiting the dSACs (probably other dSACs) or perhaps DSE at CFF-axon terminals onto these other cells. Taken together, these results suggest that dSACs can display disynaptic inhibition that is sensitive to CB_1_-R activation.

### Modulation of CFF-EPSCs and IPSCs in GCs

**Figure 6:**
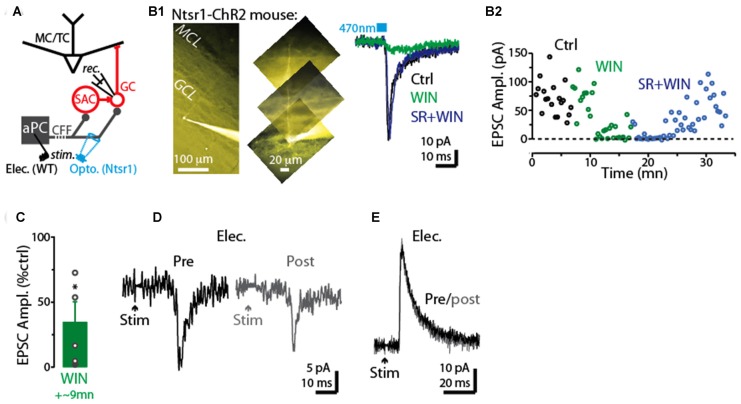
CB_1_-R-mediated modulation of CFF-EPSCs in GCs. **(A)** Schematic illustrating protocol for measuring CFF-EPSCs in GCs. CFF-EPSCs were evoked by either light stimulation in ntsr1-ChR2-YFP mice or electrical stimulation in aPC. **(B1)** Right: light-evoked CFF-EPSCs recorded in a GC (*V*_hold_ = −75 mV) were reduced by WIN and recovered by subsequent addition of SR (in the presence of WIN). Each trace reflects average of six trials. Control and WIN traces are difficult to distinguish because they are nearly identical. Left: epi-fluorescence image of the GC, indicating the presence of an apical dendrite. **(B2)** Time plot of the peak amplitude of the light-evoked CFF-EPSC for the experiment in **(B1)** (one data point per trial). **(C)** Summary of WIN effects on CFF-EPSCs in five GCs (12 trials per cell). *p = 0.024, paired *t*-test, *n* = 5. **(D)** Example recording of CFF-EPSCs in a GC before (black; average of 6 trials) and 2.5 s after (gray) a 5-s depolarization to the GC. Note the evidence for DSE in this GC. The holding potential was −75 mV. **(E)** Example recording of CFF-driven IPSCs in a GC before (black; average of 6 trials) and 2.5 s after (gray) a 5-s depolarization to the GC. The holding potential was −45 mV.

We next examined whether CFF axon terminals with CB_1_-Rs (Soria-Gómez et al., 2014) terminate on GCs, making their CFF-EPSCs sensitive to activation of these receptors. In voltage-clamp recordings from five GCs (Figure 6A), the CB-R agonist WIN reduced the CFF-EPSCs (66 ± 16% decrease, *p* = 0.024 in paired *t*-test; −62 ± 13 pA in control vs. −15 ± 7 pA in WIN; Figures 6B1–C). In three of these GCs onto which we then co-perfused the antagonist SR compound, the effect of WIN was fully or partially reversed by SR (example in Figure 6B). In addition, in 3 of 4 GCs tested, the CFF-EPSC was reduced by a prior 5-s depolarization (to 0 mV) applied to the post-synaptic GCs (example in Figure 6D), supporting DSE at CFF-to-GC contacts. In a subset of GCs, stimulation of the CFF pathway evoked an IPSC with disynaptic characteristics likely mediated by dSACs (Pressler and Strowbridge, 2006; Boyd et al., 2012). This IPSC was not sensitive to a prior 5-s depolarization of the GC (10 ± 5% increase following depolarization, *p* = 0.27 in paired *t-test*, *n* = 5; Figure 6E), arguing against depolarization-induced suppression of the disynaptic IPSC driven by CFF stimulation (due, for example, to CB_1_-R activation at dSAC-to-GC synapses).

### Control of Disynaptic Inhibition Onto MCs

**Figure 7:**
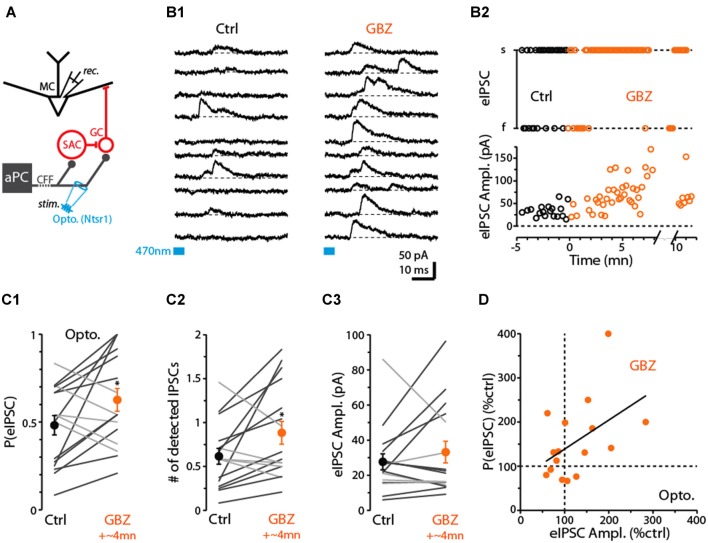
Evidence for dSAC-mediated disinhibition of Mitral cells (MCs). **(A)** Schematic illustrating protocol for measuring disynaptic (CFF axon-to-GC-to MC) IPSCs in MCs. CFF stimulation was always conducted optogenetically in slices obtained from ntsr1-ChR2-YFP mice. IPSCs were recorded at depolarized holding potentials (0 mV or −45 mV). **(B1)** Consecutive current traces recorded in a MC at 0 mV. Under control conditions (left), disynaptic IPSCs were only occasionally observed, but a low concentration of gabazine (0.5 μM GBZ) enhanced the probability of the IPSCs. Light-stimulation was conducted by focusing the 40× objective in the GCL, 280 μm from the MCL. **(B2)** Time plots for the experiment in **(B1)**, showing increases in the success-rate of light-evoked IPSCs due to low GBZ (top) and increased amplitude of the IPSCs (bottom). Each data point reflects one trial. **(C)** Summary of low GBZ effects on the probability of evoking an IPSC **(C1)**, the number of evoked IPSCs **(C2)**, and the amplitude of the IPSCs **(C3)**. Plots reflect recordings from 16 MCs (averages of 24 trials per cell). MCs in which low GBZ increased the IPSC probability are shown with black lines in all plots; other MCs shown with light gray lines. *in **(C1)** *p* = 0.040, paired *t*-test, *n* = 16; *in **(C2)** *p* < 0.05, paired *t*-test, *n* = 16. **(D)** Low GBZ-effects on the probability of evoking an IPSC plotted as a function of the effect on IPSC amplitude. Line: linear regression fit, *R*^2^ = 0.23, *p* = 0.06.

Thus far, we have provided evidence that CB_1_-Rs can downregulate CFF-EPSCs in GCs, which inhibit MCs, as well as CFF-EPSCs in dSACs that may act to disinhibit MCs through their GABAergic connections onto GCs. These dual effects raise the question of what the net effect of CB_1_-R activation would be on GC-mediated inhibition of MC/TCs. In our final studies, we examined this issue using recordings of inhibitory currents in MCs in response to CFF stimulation (Figure 7A; Balu et al., 2007; Boyd et al., 2012; Markopoulos et al., 2012; *V*_hold_ = −45 mV or 0 mV). In optogenetic experiments performed in ntsr1-ChR2-YFP mice, we found that brief (5 ms) light stimulation in the GCL at 250–300 μm from the soma of the test MCs evoked outward currents (Figure 7B1) that reversed polarity at −60 ± 1 mV (*n* = 18) near the chloride reversal potential in these experiments (–63 mV), consistent with GABA_A_ receptor-mediated IPSCs. The IPSCs were eliminated by the glutamate receptor antagonists NBQX (25 μM) + D-APV (50 μM; 82 ± 4% reduction, *p* = 0.02 in paired *t*-test, *n* = 6), occurred 7.0 ± 0.3 ms (*n* = 18) after stimulation, and a small EPSC was frequently observed prior to the IPSC (in 26 out of 37 MC recordings). These features confirm the disynaptic (CFF axon-to-GC-to-MC) nature of the MC IPSCs.

Before we assessed the effect of CB_1_-R activation on the disynaptic IPSC in MCs, we first wanted to investigate whether dSACs can in fact function in a disinhibitory capacity. While dSACs clearly contact GCs (Pressler and Strowbridge, 2006; Eyre et al., 2008), no studies have directly examined whether dSACs can control the amount of inhibition that GCs provide onto MCs following CFF activation. To address this issue, we measured MC IPSCs in response to CFF axon stimulation before and after application of a low concentration of gabazine (GBZ; 0.5–1.0 μM). Our design was to partially suppress dSAC-to-GC inhibition while still being able to observe GC-to-MC inhibition. A GBZ-induced increase in the probability and/or frequency of MC IPSCs evoked by CFF stimulation would provide evidence for dSAC-mediated disinhibition. Indeed, under conditions in which the intensity of the light stimulus was set to “threshold” levels for evoking MC IPSCs under control conditions (failure rate = 52 ± 6%), application of low GBZ increased both the probability of evoking an IPSC (by 55 ± 21%, *p* = 0.040 in paired *t*-test, *n* = 16; Figures 7B1–C1) and also increased the number of evoked IPSCs (by 72 ± 26%, *p* < 0.05, *n* = 16; in 50 ms window following the stimulus; Figure 7C2). In addition, MCs that displayed a low-GBZ-induced increase in IPSC probability tended also to have an increased peak amplitude for the evoked IPSC (Figures 7B2,C3,D; linear regression of GBZ-effects on IPSC amplitude vs. probability: *R*^2^ = 0.23, *p* = 0.06). Increased amplitudes would be expected if low GBZ increased the probability of exciting synchronized GCs (Schoppa, 2006a). These results taken together provide evidence that dSAC-to-GC inhibition can suppress GC-to-MC inhibition. It should be noted that we could not be certain that the low-GBZ-induced increases in MC IPSCs reflected an impact of the drug on dSAC-to-GC transmission specifically evoked by CFF stimulation; effects could have been due to changes in the baseline excitability of GCs. Arguing against this however was that the frequency of spontaneous IPSCs (sIPSCs) in MCs, which should reflect baseline excitability of GCs, was not significantly altered by low-GBZ (13 ± 11% frequency increase, *p* > 0.1, *n* = 16).

**Figure 8:**
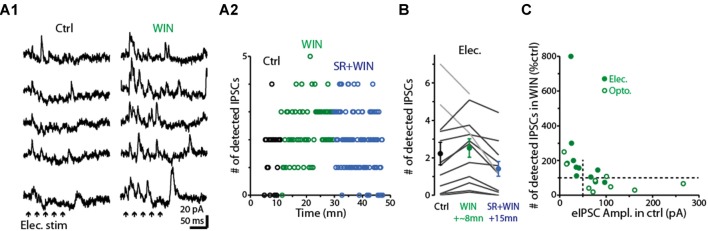
Activation of CB_1_-Rs enhances small disynaptic IPSCs in MCs. **(A1)** Consecutive trials of disynaptic inhibitory responses in a MC (*V*_hold_ = −45 mV) evoked by CFF stimulation under control conditions (left) and in the presence of WIN (right). CFF axons were activated using electrical stimulation in aPC (5 stimuli, 40 Hz; indicated by black arrows). **(A2)** Time-course for the experiment in **(A1)** showing that WIN increased the number of detected IPSCs following stimulation (assessed over 250 ms-window), and that this effect was reversed by co-application of the CB_1_-R antagonist SR. Each data point reflects one trial. **(B)** Summary of WIN and SR effects on the number of MC IPSCs evoked by electrical stimulation in aPC (*n* = 12; 12–24 trials per MC under each condition). Note the increase due to WIN in 10 MCs (dark lines) and the reversal in the same cells by co-application of WIN+SR. Circles: mean ± SEM. Responses were evoked by bursts of 40 Hz (as seen an **A1**) or 100 Hz electrical stimuli. **(C)** WIN effects on the number of disynaptic IPSCs as a function of the amplitude of the evoked IPSC (eIPSC) under control conditions. MC recordings in which IPSCs were measured following electrical stimulation of aPC (as in **A1**; dark symbols) or optogenetic stimulation (open symbols) in ntsr1-ChR2-YFP mice are shown. Note that weak eIPSCs were consistently enhanced by WIN, while strong eIPSCs were most commonly reduced by WIN. Vertical dashed line at 50 pA reflects the cut-off used in the analysis in which WIN effects were determined by IPSC size (see main text). All IPSC recordings in the illustrated experiments were conducted using a −45 mV holding potential.

Having determined that dSACs are capable of disinhibiting MCs, we next examined the effect of the CB-R agonist WIN (10 μM) on the strength of MC inhibition evoked by CFF axon stimulation (Figure 8). Is the effect of CB-R activation stronger at CFF axon-to-dSAC synapses than at CFF axon-to-GC synapses, in which case WIN should increase MC IPSCs, or are the effects of receptor activation opposite to that, in which case WIN should decrease the IPSCs? Interestingly, we found that the results depended on the method of CFF stimulation. When CFF axons were stimulated optogenetically, as in the low-GBZ experiments, WIN (10 μM) could both increase or decrease the IPSCs and overall had no effect (15 ± 26% decrease, *p* > 0.1, *n* = 9; see summary in Figure 8C). However, with electrical stimulation in aPC (a 40/100 Hz burst of 5/10 100 μs pulses in these experiments), WIN elicited clear increases in the number of evoked IPSCs in 10 of 12 MCs (increase in the 10 MCs = 65 ± 14%, *p* < 0.005, *n* = 10; Figures 8A1,A2,B). In addition, subsequent application of the CB_1_-R antagonist SR mainly reversed the WIN effect (52 ± 10% decrease in IPSC number from WIN level, *p* < 0.001, *n* = 10; Figures 8A2,B). The WIN-induced increase in MC IPSCs would be consistent with a dominant role for CB_1_-R-mediated down-regulation of CFF axon-to-dSAC transmission, but clearly, based on the optogenetic results, this was not always the case.

Further analysis suggested that the dependence of the WIN-effect on the stimulation method may have at least in part reflected the effectiveness of the stimuli in driving GC-to-MC inhibition. The MC IPSCs driven by optogenetic stimulation were generally larger than those due to electrical stimulation, and, in the few instances in which WIN increased light-evoked IPSCs, the control IPSCs were small (Figure 8C). In addition, when we separated our complete data-set by control IPSC amplitude rather than stimulation method, WIN induced a robust 161 ± 66% increase (*p* < 0.01, *n* = 10) in the number of IPSCs in MCs with small IPSCs (<50 pA peak amplitude) and, in fact, decreased the IPSC number (by 27 ± 11%, *p* = 0.042 in paired *t*-test, *n* = 11) in MCs with larger IPSCs (>50 pA). The WIN-induced decreases in the MC IPSCs would be consistent with CB_1_-R-mediated down-regulation of CFF axon-to-GC transmission over-riding receptor-mediated down-regulation of CFF axon-to-dSAC transmission. Altogether, these data suggest that CB_1_-R activation can bidirectionally influence the degree of GC-to-MC inhibition, with effects that depend on the properties of local network activation (see “Discussion” section).

## Discussion

In the present study, we evaluated the role of cannabinoid receptors in modulating CFF to the OB, focusing mainly on actions within the GCL. We found in bulb slice recordings that CB_1_-Rs function at CFF axon terminals that target a number of different cell-types and that this ultimately can result in altered inhibition of MCs. The ability of CB_1_-Rs to gate inhibition on MCs could be important for odor-evoked inhibition and/or neural synchrony.

### CB_1_-R-Mediated Presynaptic Modulation at CFF Terminals

CB_1_-Rs are expressed at excitatory and inhibitory axon terminals of many neuron types throughout the brain, where they inhibit transmitter release by reducing presynaptic calcium currents and/or promoting activation of potassium channels (Araque et al., 2017). In most cases, presynaptic CB_1_-Rs can be retrogradely activated by anandamide/2-arachidonylglycerol released from postsynaptic or nearby neurons undergoing strong depolarization, leading to DSE or DSI. Here, building on prior evidence that CB_1_-Rs are expressed at CFF axon terminals (Soria-Gómez et al., 2014), we have found that these receptors can also mediate a reduction of glutamate release at CFF terminals and can be activated when postsynaptic cells undergo strong depolarization. We most extensively studied CB_1_-R-mediated presynaptic inhibition at CFF axon contacts onto GABAergic dSACs (Boyd et al., 2012). In recordings from dSACs, the CB-R agonist WIN 55,212-2 (WIN) drastically reduced the CFF-EPSC with an effect that was reversed by the CB_1_-R antagonist SR-141716A (SR). Moreover, the decrease in the EPSCs was associated with an increase in the PPR, an effect that is commonly taken as evidence for changes in presynaptic probability of release. DSE was manifested as a reduction in the EPSC elicited by a prolonged 5-s depolarization applied to dSACs, corresponding changes in the PPR, as well as blockade of the depolarization-induced effects by the antagonist SR.

One caveat in the analysis of DSE was related to the nature of the depolarizing stimulus applied to dSACs, which was a 5-s depolarization to 0 mV. Prior studies have shown that the most numerous class of dSACs, the Blanes cells (Eyre et al., 2008), can undergo long-lasting depolarizations and spike activity that can last at least that long (Pressler and Strowbridge, 2006), but a stimulus to 0 mV may be unnaturally strong. Further studies are thus needed to establish the requirements for endocannabinoid release from dSACs. In addition, a concern with the experiments analyzing the effect of SR on DSE was that the SR-containing solution also contained the agonist WIN in some of the recordings. This however does not significantly impact our conclusion that presynaptic CB_1_-Rs mediate DSE at CFF-to-dSAC synapses, for a number of reasons. First, we were able to observe clear elimination of DSE in four experiments in which SR was applied alone. Also, even with a mixture solution that contains both SR and WIN, elimination of DSE due to either drug would be through a CB_1_-R mediated mechanism, antagonism of endocannabinoid binding to the receptors due to SR or occlusion of binding due to WIN. It should also be pointed out that at the concentrations of SR and WIN that we used (both at 10 μM) SR was likely antagonizing WIN binding nearly completely when the mixture was applied. This would account for the fact that, in the initial analysis of the drug effects on the CFF-EPSCs (Figure 1D; in the absence of prolonged depolarization), SR fully reversed the effect of WIN.

While a major focus of our study was on CFF contacts onto dSACs, we also found that CB_1_-R-mediated effects were relatively widespread. For example, WIN significantly reduced excitatory transmission from CFF axons onto GCs (Shipley and Adamek, 1984; Balu et al., 2007; Laaris et al., 2007; Matsutani, 2010; Boyd et al., 2012; Markopoulos et al., 2012). DSE also occurred across subtypes of dSACs differentiated by location and morphology, including dSACs in the GCL that can contact GCs (Pressler and Strowbridge, 2006; Eyre et al., 2008) as well as dSACs in the IPL that send inhibitory projections to the GML (Eyre et al., 2008; Burton et al., 2017). We even obtained evidence for DSI at inhibitory synapses onto dSACs, potentially reflecting inputs from other dSACs. Our results suggesting CB_1_-R actions at several sites in the more inner regions of the bulb add to prior results indicating that CB_1_-R-mediated DSI can occur at synapses in the GML between periglomerular cells and external TCs (Wang et al., 2012). One notable synaptic connection where CB_1_-R-mediated DSE was not observed in our study was at feedforward excitatory contacts from TCs/MCs onto dSACs (Burton and Urban, 2015). In dSAC recordings in which clear DSE occurred for CFF inputs, there was no suppression of feedforward excitation. Mechanistically, the specificity of CB_1_-R-mediated synaptic suppression, occurring only for feedback excitation, could have been because presynaptic glutamate release sites on TCs/MCs lack CB_1_-Rs or because TC/MC-to-dSAC contacts are distant from the sites of cannabinoid release on dSACs.

### Modulation of MC/TC Inhibition by CFF Pathways

Studies conducted *in vivo* in recent years have begun to provide information about the function of CFF inputs into the OB, especially for those inputs that terminate on GCs. For example, activating CFF axons can enhance synchronized gamma frequency oscillations in the bulb (Boyd et al., 2012), which are well-known to be mediated by GC-to-MC connections (Rall and Shepherd, 1968; Mori et al., 1999; Lagier et al., 2004; Galán et al., 2006; Schoppa, 2006b; Boyd et al., 2012; Fukunaga et al., 2014). Activation or suppression of CFF inputs from aPC can also alter odor-evoked inhibition of MCs (Boyd et al., 2012; Otazu et al., 2015), potentially through changes in GC activity. The role of CFF contacts onto dSACs, which could mediate disinhibition of MC/TCs through their contacts on GCs (Pressler and Strowbridge, 2006; Eyre et al., 2008; Boyd et al., 2012), have in contrast received relatively little attention beyond the observation that CFF axons target dSACs (Boyd et al., 2012).

Here we have addressed the function of CFF axon-to-dSAC connections in two ways. First, we have provided evidence that dSAC-to-GC inhibition can impact the level of inhibition onto MCs elicited by CFF activation, i.e., that dSACs can function in a disinhibitory capacity. This was based on the effects of a low concentration of gabazine on disynaptic inhibitory responses in MCs evoked by CFF stimulation (Figure 7). Prior studies have provided evidence for dSAC-mediated disinhibition of MCs (Nunes and Kuner, 2015), but have not focused on responses driven by CFF axons as we did. Second, we found that the CB-R agonist WIN could increase inhibitory responses in MCs evoked by CFF stimulation, consistent with the level of GC-to-MC inhibition being regulated by CB_1_-Rs at CFF-to-dSAC synapses. Interestingly, the WIN effect in these experiments varied in a manner that depended on the magnitude of MC inhibition. WIN consistently increased MC IPSCs when inhibition levels were low, but most commonly decreased the IPSCs when inhibition was large. We propose that these bidirectional effects reflected the relative levels of activation of GCs vs. dSACs by CFF stimulation across the different experiments. Low levels of GC-to-MC inhibition (small MC IPSCs) would correspond to when dSACs were well-activated and/or if GCs were only weakly excited and hence susceptible to being inhibited by dSACs. Under this condition, WIN-induced suppression of CFF-to-dSAC transmission could exert a strong enhancing effect on GC-to-MC inhibition. High levels of GC-to-MC inhibition (large MC IPSCs) in contrast would correspond to when CFF axons effectively stimulated more GCs and/or each GC was more strongly excited. Here, a WIN-induced decrease in CFF axon-to-GC transmission would either cancel out the WIN effect on CFF axon-to-dSAC transmission or override it. Notably, in the *in vivo* study by Soria-Gómez et al. (2014), exogenous WIN application enhanced MC/TC spike activity evoked by optogenetic stimulation of CFF axons in the GCL, an effect that may have reflected CB_1_-R-mediated down-regulation of CFF axon-to-GC transmission. The WIN-induced decreases in the larger MC inhibitory responses that we observed would reflect a similar effect.

In addition to there being a relationship between the effect of WIN and the amplitude of the MC IPSC, we found that the MC IPSC amplitude was roughly related to the method of stimulating CFF axons. Electrical stimulation typically drove smaller IPSCs than optogenetic stimulation, a relationship which made the WIN effect on MC IPSCs dependent on the stimulation method. We speculate that the difference in the size of the IPSCs evoked by the two stimulation methods reflected the spatial breadth of CFF axon activation. Electrical stimulation in aPC likely resulted in CFF axons that were excited over widespread areas of the bulb, which could have been a relatively effective stimulus for exciting dSACs with large dendritic arbors spanning hundreds of microns (Eyre et al., 2008). Optogenetic stimulation involved the application of spatially restricted light pulses in the GCL through a 40× objective (over a ~100-diameter area), which may have been more effective at exciting small GCs.

How would the release of natural endocannabinoid compounds alter MC/TC inhibitory responses during odor-evoked responses? Clearly a key factor would be the depolarization status of dSACs and GCs, since the release of endocannabinoids typically requires prolonged depolarization (Kreitzer and Regehr, 2001; Ohno-Shosaku et al., 2002; Kano et al., 2009). Such depolarizations could be driven by either intrinsic conductances (Pressler and Strowbridge, 2006) or perhaps be the result of an odor stimulus during prolonged sniffing. CB_1_-Rs at CFF terminals could bidirectionally control the level of odor-evoked MC/TC activity as a function of how effectively an odor depolarized the local population of GCs and dSACs resulting in endocannabinoid release.

## Author Contributions

FP and NES: design of experiments, interpretation of results and writing of manuscript. FP: acquisition and analysis of data.

## Conflict of Interest Statement

The authors declare that the research was conducted in the absence of any commercial or financial relationships that could be construed as a potential conflict of interest.
